# Illustrations of the Comparative Anatomy of the Nervous System

**Published:** 1836-04-01

**Authors:** 


### Illustrations of the Comparative Anatomy of the Nervous System—Part I. By *Joseph Swan.* London: Longman and Co. 1835.

Mr. Swan's merits, as a most assiduous and minute dissector of the nervous system, have been so well known to the profession and the scientific public, ever since the council of the College of Surgeons adjudged their prizes to him for his dissections of this system, that his name will, we doubt not, prove a sufficient recommendation to the work before us. Without the least desire to detract one iota from the well-earned reputation of our author, yet we must, on the present occasion, take the opportunity of recording our sentiments respecting the work on which that reputation is principally founded.

The "Demonstration of the Nerves of the Human Body" is doubtless a work reflecting great credit upon the author for careful and industrious dissection, but we fear it has not yet effected much for the science of physiology. Undertaken, as it was, at so important an æra in neurological science, when the beautiful discoveries of Bell and Magendie had not long been given to the world, and when numerous theories, not meriting to rank with discoveries, were put forth by these and other authors, we cannot but regret that its author did not, more than he appears to have done, direct his attention to what had been done by other anatomists, with the view of examining and correcting their various statements, and reconciling their discrepancies. In this way, and having carefully weighed the doctrines and theories in nervous physiology, how much good might not have been effected by such an undertaking. We write this in especial reference to the nature and functions of many of the cerebral nerves, and to Sir C. Bell's doctrine of a respiratory system, towards the satisfactory settlement of which questions, anatomical research must yet contribute much. We will only stop, however, to mention the otic ganglion, described by Arnold ten years since.* At the same time we must admit that probably Mr. Swan deemed it better simply to state the facts he had himself made out.

The question as to whether the connexion of the sympathetic with the spinal nerves be with their motiferous or sensiferous nerves, or with both, has not engaged his attention, though highly important; and, on the Continent, employing the labours of Müller, Retzius, Mayer, Wützel, and Scarpa, forming, perhaps, one of the very last subjects to which that great anatomist directed his attention. Many other, too, of the more recondite, but not less important questions in this department of anatomy, might be mentioned as not particularly noticed by Mr. Swan.

We hesitate not to state, that it will not be until we have a still more minute dissection of the cerebral nerves than has yet appeared, especially as regards the connexion and anastomosis of the nervous branches, that we shall be able fully to comprehend the functions of many of them, and enabled to perceive that their arrangement is the same as that of the spinal nerves. The appearance of Arnold's "Icones Nervorum Capitis," does not even altogether satisfy this desideratum, though making considerable advance.

The following extracts from our author's preface to his present work, will explain the views he had in its publication.

"The author having had frequent occasions for referring to comparative anatomy, during the course of his publications on the nervous system, he conceived that representations of his dissections might interest many, and especially assist those who were about to enter on similar pursuits." "It appeared probable that this intention would be in a great measure answered, by selecting

---

* Dissertatio Inauguralis, de Parte Cephalica Nervi Sympathetici in Homine. Fredericus Arnold. Quarto. Heidelbergæ, 1825.

such examples as would explain the scheme on which the nervous system is generally founded."

"It is desirable that researches by actual dissection should be encouraged as much as possible, as thus alone can the prospective advantages of science be consulted. If books be entirely confided in, all improvement must stop. These ought, therefore, to be esteemed as mere helps towards that perfection, which can only be approached by the active perseverance of individuals through progressive ages."

In the latter observations of our author we fully agree; but we fear that he has somewhat lost sight of the important fact, that the converse statement is no less true—that all improvement must stop, if we do not acquaint ourselves with what others have done and are doing, in that particular branch of enquiry to which we may devote ourselves.

The part of the work before us contains seven plates, drawn by West, and beautifully engraved by Finden, and embracing this part of the anatomy of the crab, the lobster, the leech, the earth-worm, the centipede, the slug, and the whelk, amongst invertebrate animals, and that of the cod in fishes. The delineations are very distinct, and appear to possess the important requisite of minute accuracy. We can hence speak in the highest terms of this Number, and recommend it to our anatomical friends. We cannot, however, do otherwise than point out what we regard as slight defects in its execution.

These may be divided into two orders—those of omission and those of commission. We will devote a few moments to the consideration of each.

Our author does not allude to the recent valuable researches of Mr. Newport,* on the Development and Structure of the Nervous System of articulated Animals. These researches not only confirm the previous statements of Swammerdam, Lyonet, Herold, Meckel, Steviranus, Müller, Dufour, Straus Durckheim, Weber, Audouin, and Edwards, and others, on many points in the comparative anatomy of the nervous system not referred to by Mr. Swan, but likewise have added much that is altogether new to our knowledge of this intricate, but highly-interesting subject. Mr. Newport shews, in the plates appended to his papers, and asserts, the existence of three distinct tracts in the ganglionic nervous cord of the articulata, each of which is engaged in giving origin to distinct nerves, supposed to possess distinct functions, analogous to the anterior and posterior roots of spinal nerves in vertebrate animals—with the hypothetical respiratory nerves of Sir C. Bell. On this point, we hold that our author might advantageously have stated the result of his individual researches, whether confirmatory of, or in opposition to, the statements of Mr. Newport. We should have been much pleased with the confirmation of these interesting researches, which, coming from Mr. Swan, would have been so truly valuable. We trust, however, that in the future Numbers of the work, this error may be repaired, and are desirous of pressing the matter closely on Mr. Swan's attention.

Audouin and Edwards, in their researches on the anatomy of the crustacea,† have satisfactorily shewn, that the nervous system is formed on the same general model in those genera having a lengthened and those with a

* Philosophical Transactions for 1832 and 1834.
† Annales des Sciences Naturelles, tome xix. 181.

circular form, as, for example, the lobster and crab; that in their early stage of development the nervous system of each consists of a double chain of ganglia; that these ganglia approximate to each other as the legs become developed; and that whilst they remain permanently distinct in the lobster, supplying separately the extremities, in the crab they gradually meet, and become blended in one nervous ring, from which diverge the several pairs of nerves to the feet and tail; thus explaining why there is not in the tail of the latter animal, to use the words of our author, "any appearance of ganglia, as in the tail of the lobster, scorpion, &c."

The plates which illustrate the nervous system of the lobster are excellent, and, so far as they go, fully confirm Mr. Newport's researches. The ganglionic enlargements are distinctly seen on the ventral surface of the cord, whilst the motor tract is as plainly seen passing over them on the dorsal aspect, without entering into their formation. Finally, in the inter-ganglionic spaces are seen the more delicate "superadded, transverse, or respiratory nerves" of Newport.

We need not stop further, with respect to the contents of the 5th plate, than to observe, that the 5th, 6th, and 7th figures, representing the nervous system of the slug and whelk, exemplify this part of the organization of these gasteropodous molluscæ, with a fidelity and minuteness hitherto, we believe, unequalled. These observations apply likewise, if possible perhaps, with still greater propriety, to the 6th plate, in which we have a most beautiful representation of the ganglionic nervous system of the cod. The great depth of shading, however, whilst it adds to the beauty of the engraving as a work of art, detracts somewhat from its utility, by obscuring some of the minuter nervous ramifications.

The interesting disposition of the spinal nerve of fishes, which our author points out in the cod, is so remarkable, that we extract his description of it.

"Each anterior and posterior bundle issuing from the spinal chord is divided into two branches; one branch from the anterior bundle is joined by one from the posterior, and passes forwards to the muscles and skin on the anterior part of the spine; the other branch of the anterior bundle, after it has communicated with the posterior branch passing forward, passes backward, and is joined by the second branch of the next posterior bundle, to terminate on the muscles and skin on the posterior part of the spine. Neither of the branches of the posterior bundle forms a ganglion." 26.

We are at issue, however, with our author as to his last statement. We well remember, when first allured into the study of comparative anatomy, and having no other work in our hands than the meagre production of Fyfe, being sadly puzzled to reconcile his statement of the absence of the intervertebral ganglia in fishes, with the doctrine of the "unity of composition," of which we were then, as now, most ardent disciples; we are now however satisfied that the statement is erroneous. On this head, we find the following explicit statement by E. H. Weber, in his very valuable work, "Anatomica Comparata Nervi Sympathici."

"Omnia enim vertebrata, mammalia, aves, amphibia et *pisces* gangliis spinalibus instructa sunt, equibus nervi spinales auctiores, quam intraverunt prodeunt. Si nonnulli anatomici, et in his Arsuky, negaverunt, pisces gangliis spinalibus instructor esse, id duplici causa accidisse arbitror, primum, quia fortasse majore ganglia expectaverunt quam adessc possunt, propter exiguitatem nervorum spinalium, deinde quia crassitiem nervorum spinalium cum exiguitate radicum non satis accurate comparaverunt."

We hope our author will see the importance of examining this point more minutely, before the statement is again reiterated.

For the present, we part with Mr. Swan, hoping to see the present part of his "Illustrations" quickly followed by numerous successors, each of which, we trust, will rival its predecessor in faithful anatomical detail, with especial reference to all dissected points, and particularly to those bearing upon the physiology of the nervous system. Every anatomical student should possess this work.

